# Diseases of Metabolism and Nutrition

**Published:** 1904-12

**Authors:** 


**Diseases of Metabolism and Nutrition.** By Prof. Dr. CARL VON NOORDEN. Part IV.—*Acid Auto-intoxication*, pp. 80. Part V.—*Saline Therapy*, pp. 89. Bristol: John Wright & Co. 1904.

This series of clinical treatises on the pathology and therapy of disorders of metabolism and nutrition has already been noticed previously—vol. xxi., p. 159.

It has long been well known that the intestine is a constant source of poisons, which under certain conditions cause grave alterations in the principal organs and functional troubles, among which those of the nervous system occupy a prominent place. The recent investigations of von Noorden tend to show that (1) there are numerous forms or manifestations of self-poisoning; (2) the acid forms are amongst the gravest of them; and (3) those special perversions of metabolism resulting in the excessive production of oxybutyric acid, diacetic acid, and acetone, which so greatly endanger diabetics and also complicate at times other diseases more or less seriously, are of the utmost practical importance. The work of Bouchard and his pupils has been fully confirmed from German observers, and it has been found that such intoxications may be associated with diarrhœa, or more commonly with constipation, and are to be remedied by modifications of the diet, by sterilisation of the intestine, by drugs, and by mechanical cleansing of the alimentary tract. Much valuable work as to the origin of acetone bodies, whether diabetic or otherwise, has been done, and this book gives a full account of the pathological and therapeutic considerations of diabetic acidosis.

The therapy of the saline waters has hitherto been almost entirely empirical, but recently Dr. von Noorden, with the assistance of Dr. Carl Dapper, of Bad Kissingen, has endeavoured to place balneological methods of treatment on a more scientific basis.

